# Renal Programming by Transient Postnatal Overfeeding: The Role of Senescence Pathways

**DOI:** 10.3389/fphys.2020.00511

**Published:** 2020-05-25

**Authors:** Christian Juvet, Benazir Siddeek, Catherine Yzydorczyk, Catherine Vergely, Katya Nardou, Jean-Baptiste Armengaud, Mohamed Benahmed, Umberto Simeoni, François Cachat, Hassib Chehade

**Affiliations:** ^1^Division of Pediatrics, Developmental Origins of Health and Disease (DOHaD) Laboratory, Woman-Mother-Child Department, Centre Hospitalier Universitaire Vaudois and University of Lausanne, Lausanne, Switzerland; ^2^Division of Pediatrics, Woman-Mother-Child Department, Centre Hospitalier, Universitaire Vaudois and University of Lausanne, Lausanne, Switzerland; ^3^Inserm UMR866, Laboratoire de Physiopathologie et Pharmacologie Cardio-Métaboliques (LPPCM), Faculties of Medicine and Pharmacy, University of Burgundy, Dijon, France; ^4^Division of Pediatrics, Pediatric Nephrology Unit, Woman-Mother-Child Department, Centre Hospitalier Universitaire Vaudois and University of Lausanne, Lausanne, Switzerland

**Keywords:** programming, overnutrition, postnatal overfeeding, kidney, chronic kidney disease, developmental origins of health and disease

## Abstract

**Background:**

Early nutrition influences the risk of chronic kidney diseases (CKDs) development in adulthood. Mechanisms underlying the early programming of altered renal function remain incompletely understood. This study aims at characterizing the role of cell senescence pathways in early programming of CKD after transient postnatal overfeeding.

**Materials and Methods:**

Reduced litters of 3 mice pups and standard litters of 9 mice pups were obtained to induce overfed animals during lactation and control animals, respectively. Animals were sacrificed at 24 days (weaning) or at 7 months of life (adulthood). Body weight, blood pressure, kidney weight, and glomerular count were assessed in both groups. Senescence pathways were investigated using β-Galactosidase staining and Western blotting of P16, P21, P53, P-Rb/Rb, and Sirtuin 1 (Sirt1) proteins.

**Results:**

Early overfed animals had a higher body weight, a higher blood pressure at adulthood, and a higher glomerular number endowment compared to the control group. A higher β-Galactosidase activity, a significant increase in P53 protein expression (*p* = 0.0045) and a significant decrease in P-Rb/Rb ratio (*p* = 0.02), were observed at weaning in animals who underwent early postnatal overfeeding. Protein expression of Sirt1, a protective factor against accelerated stress-induced senescence, was significantly decreased (*p* = 0.03) at weaning in early overfed animals.

**Conclusion:**

Early postnatal overfeeding by litter size reduction is associated with increased expression of factors involved in cellular senescence pathways, and decreased expression of Sirt 1 in the mouse kidney at weaning. These alterations may contribute to CKD programming after early postnatal overfeeding.

## Introduction

An upward trend in the prevalence of non-communicable diseases (NCDs) with developmental origins, including chronic kidney disease (CKD) and arterial hypertension is observed worldwide (Hanson et al., 2011; Barouki et al., 2012). Converging epidemiological studies across a range of populations over the life course and experimental studies in animals have shown that the environment during fetal and early postnatal life affects the risk of developing chronic diseases later in life (Martin et al., 2005; Dotsch et al., 2012; Adair et al., 2013; Habbout et al., 2013b). This concept, also referred to as Barker’s hypothesis, is known as Developmental Origins of Health and Disease (Barker et al., 1989; Hanson and Gluckman, 2008).

The relationship between nutrition and CKDs is well established. In the Framingham Offspring cohort (Fox et al., 2004) and in health screening programs in the United States (Hsu et al., 2006) and Japan (Iseki et al., 2004), baseline body mass index (BMI) was significantly and positively correlated to a lower glomerular filtration rate (GFR). In a New York clinical-pathologic study, the proportion of all renal biopsies that displayed obesity-related kidney damage, in particular focal segmental glomerulosclerosis (FSGS), increased 10 folds from 1986 to 2000 (Kambham et al., 2001). In both human and animal studies, early postnatal overfeeding and excessive weight gain have been associated with adverse outcomes in the vascular and renal systems in adulthood (Boubred et al., 2007, 2009; Alcazar et al., 2012; Yim et al., 2012, 2013, 2014; Adair et al., 2013). We and others showed in previous studies that postnatally overfed rats had a higher systolic blood pressure (SBP), proteinuria, increased glomerular number, smaller glomerular volume, and more FSGS at adulthood compared to control animals (Boubred et al., 2007). Similarly, an increase in albuminuria, and FSGS has been observed in postnatally overfed rats at 3, 6, and 12 months of age (Yim et al., 2013, 2014).

However, while the renal consequences of postnatal overfeeding induced by litter size reduction have been well described (Boubred et al., 2007; Yim et al., 2013, 2014), the underlying molecular mechanisms remain incompletely understood. Several pathway alterations, including the renin angiotensin system, have been described (Juvet et al., 2018). However, the pathways leading to cellular senescence have not yet been studied in this context.

Senescence pathways are known to play different roles in the kidney during the life course. During kidney development, senescence pathways participate to the regression of transitory structures, such as the mesonephros (Da Silva-Alvarez et al., 2019). In this context, senescence pathways display some specificities compared to senescence later in life, as this subtype of senescence seems to be independent of P53. Furthermore during development, P53, was shown to be important for physiological nephrogenesis (Song et al., 2018). At adulthood, activation of senescence pathways occurs in a different context, as accelerated, stress-induced senescence has been shown to play a major role in renal aging and pathology, such as glomerulosclerosis and fibrosis (Sturmlechner et al., 2017).

Furthermore, we recently demonstrated that early postnatal overfeeding leads to modifications of senescence pathways and Sirt-1 expression in the liver (Yzydorczyk et al., 2017).

In this study, we aim at characterizing the role of cell senescence pathways in early programming of renal alterations after transient postnatal overfeeding.

## Materials and Methods

### Animal Model

This study was conducted in accordance with the 2010/63/EU directive of the European Parliament and the “Guide for the Care and Use of Laboratory Animals” published by the US National Institutes of Health (NIH Publication No. 85–23, revised 1996). The study protocol was approved by the ethics committee of the university that housed the animal model (Comité d’Ethique de l’Expérimentation Animale, Université de Bourgogne, Dijon, France, protocol agreement number: 3710).

Female adult C57BL/6 mice were caged with male mice in a proportion of 2:1 for mating. During pregnancy and lactation, female mice were housed individually with an appropriately enriched environment, and fed a standard *ad libitum* chow diet with free access to water. On the third day of life, male pups were randomly cross-fostered between mothers in either reduced litters (3 pups) to induce overfeeding, or standard litters (9 pups) for the control group. Two consecutive series of reproduction were used, corresponding to 12 different litters. Each litter included pups from one to six different dams. Experiments were conducted on male pups, as the effect of postnatal overfeeding on the development of renal disease was demonstrated predominantly in male pups (Boubred et al., 2007, 2009; Alejandre Alcazar et al., 2011; Alcazar et al., 2012; Yim et al., 2013, 2014, 2017). Excess pups were sacrificed by decapitation after isoflurane anesthesia. Pups were weaned on day 24, after which they had *ad libitum* access to tap water and a standard chow diet. Animals were sacrificed at 24 days (further referred to as “weaning”) or 7 months of life (further referred to as “adulthood”) by exsanguination after intraperitoneal administration of a lethal dose of pentobarbital (80 mg/kg).

### Body Weight (BW)

Offspring mice were weighed monthly, from birth until 7 months of age. Average BW at a given age was calculated in each group.

### Blood Pressure (BP)

Blood pressure was measured at 6 months of life using a validated tail-cuff method (Boubred et al., 2007, 2009; Habbout et al., 2013a; Li et al., 2016) in anesthetized animals. For each animal, 3 measurements of SBP and diastolic blood pressure (DBP) were averaged.

### Kidney Weight (KW)

Freshly harvested kidneys were weighted immediately after sacrifice. Total KW (sum of both kidney weights) is expressed relatively to BW for each animal.

### Glomerular Count

Total glomerular number was assessed by the dissection, acid maceration method as previously published and validated (Boubred et al., 2007). Whole kidneys (1 kidney per animal) were incubated in 50% hydrochloric acid for 45 min at 37°. Incubation time was adapted according to the kidney’s weight. Kidneys were then rinsed in tap water and stored at 4° overnight. Kidneys were dissociated mechanically. Tubules and glomeruli were then suspended in water. Glomeruli from three different 0.5 ml aliquots were counted in a counting chamber under the microscope in a blinded manner. The total kidney’s glomerular number endowment was calculated from the average number of glomeruli from the three aliquots.

### Histological Analysis

Kidneys were frozen at −80° and included in Tissue-Tek OCT. Five micrometers sections were used for β-Galactosidase staining (Cell Signaling Kit #9860) according to the manufacturer’s protocol to evaluate cellular senescence (Itahana et al., 2007). Briefly, frozen sections were rinsed in phosphate buffered saline (PBS), then fixed and incubated with β-Galactosidase staining solution overnight at 37°. Negative controls were performed with the same β-Galactosidase staining solution, but lacking the X-Gal substrate of β-Galactosidase. Sections were analyzed with a Nikon Ti microscope at a magnification of 20×.

### Proteins Extraction

Frozen kidneys (−80°C) were grinded and resuspended in lysis buffer prepared with RIPA buffer (Sigma Aldrich) and protease inhibitor (cOmplete^TM^). HCl was used to adjust pH to 8.0. Samples were then dislocated by sonication and centrifuged. Supernatant was removed and stored at −20°C. Protein quantification in the obtained samples was performed with a Pierce BCA protein assay kit (23225).

### Western Blotting

Samples of 30 micrograms of proteins in Laemmli buffer were suspended in NUPAGE reducing buffer and RIPA buffer. After heating at 70°C for 10 min to induce protein denaturation, samples were charged in a 4–12% Bis-Tris gel (invitrogen NP0335BOX) running gel. Migration was performed at 100 V for 2:15 h in NUPAGE MOPS SDS running buffer (NP0001). Transfer on nitrocellulose membranes 0.45 μm (Bio-Rad 1620115) was performed overnight at 30 V in NUPAGE transfer buffer (NP0006-1). Protein transfer was checked with a Ponceau Red staining (Sigma). Membranes were then blocked in 5% bovine serum albumin (BSA) dissolved in Tris Buffered Saline with Tween (TBST). Membranes were incubated overnight with primary antibody, washed in TBST and incubated 2 h with secondary antibody. The following antibodies were used at a dilution of 1/1000: Anti P16 (Abcam Ab 201980), Anti P21 (Abcam Ab 7960), Anti P53 (Cell Signaling 2527), Anti P-Rb (Cell Signaling 8516), Anti Rb (Cell Signaling 9309), and Anti Sirt1 (Cell Signaling 9475). The other following antibodies were used at a dilution of 1/2000: Anti β Actin (Cell signaling 4967), HRP linked anti rabbit antibody (Cell signaling 7074), HRP linked anti mouse antibody (Cell signaling 7076).

Chemiluminescent signal was obtained with Western pico or Western femto, depending on the proteins expression profile (Thermo Fisher Scientific 34094 and 34577). Pictures were captured with a G-Box (Syngene) and analyzed with the image J software.

### Statistical Analysis

Student’s *t* test was applied to test for intergroup difference of BP and one-way ANOVA to test for intergroup difference of weight. For every other parameter, continuous values between groups were compared using the non-parametrical Mann-Whitney’s *U* test. *P* value was considered significant if <0.05, with no correction for multiple comparisons. No *post hoc* test was performed. Data was analyzed using GraphPad Prism 8.0.1.

## Results

### Body Weight

Birth weight was not significantly different between overfed and control groups. At weaning, animals exposed to early transient postnatal overfeeding had a significantly higher body weight (30%) compared to control group. This difference persisted throughout life until sacrifice at adulthood (Habbout et al., 2013a; Li et al., 2016). These results were previously published (Habbout et al., 2013a; Li et al., 2016).

### Blood Pressure

At 6 months of age, systolic, diastolic and mean arterial BP were significantly higher in animals who underwent early postnatal overfeeding compared to the control group. These results have been published previously (Habbout et al., 2013a; Li et al., 2016).

### Kidney Weight

**FIGURE 1:**
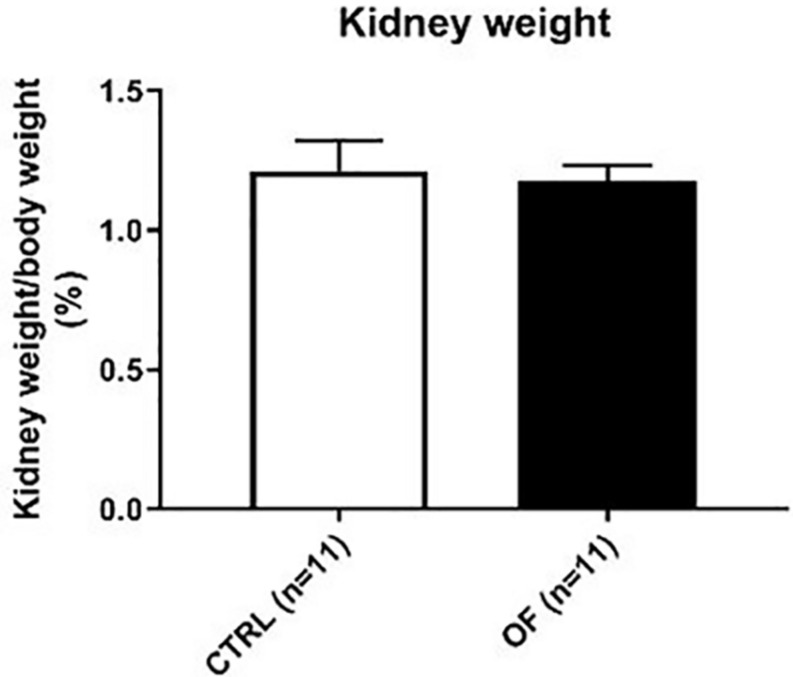
Comparison of standardized total kidney weight (sum of both kidney weights) between control (CTRL) and overfed (OF) groups at weaning. Results are expressed as means ± standard deviation (SD), *p < 0.05.

**FIGURE 2:**
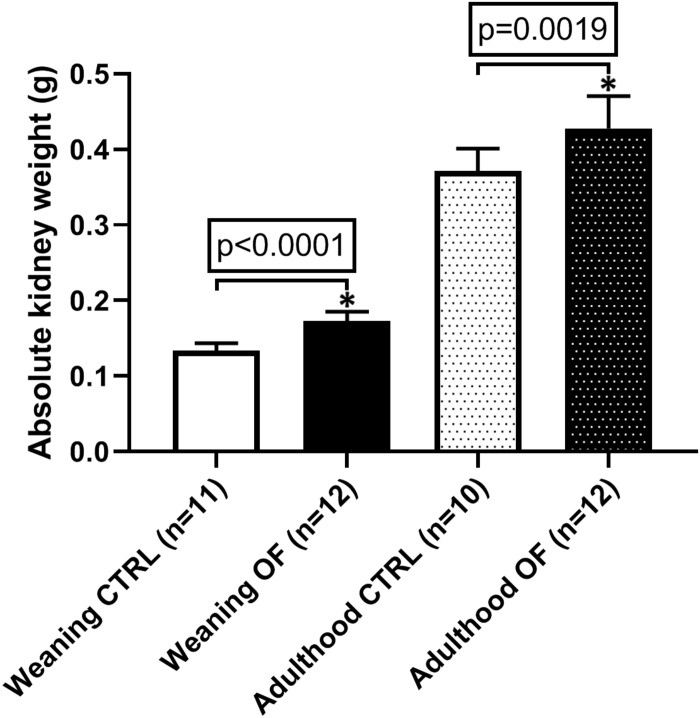
Comparison of absolute total kidney weight (sum of both kidney weights) between control (CTRL) and overfed (OF) groups at weaning and adulthood. Results are expressed as means ± standard deviation (SD), *p < 0.05.

Kidney weight was assessed at weaning. There were no differences between groups in standardized total kidney weight (i.e., sum of both kidneys expressed relatively to body weight) (*n* = 11 in each group, *p* = 0.78) (Figure 1). However, when considering absolute kidney weight, overfed animals had significantly higher total kidney weight at weaning (*n* = 11 and *n* = 12 in CTRL and OF group, respectively, *p* < 0.0001). This difference persisted at adulthood (*n* = 10 and 12 in CTRL and OF group, respectively) (Figure 2).

### Glomerular Number

**FIGURE 3:**
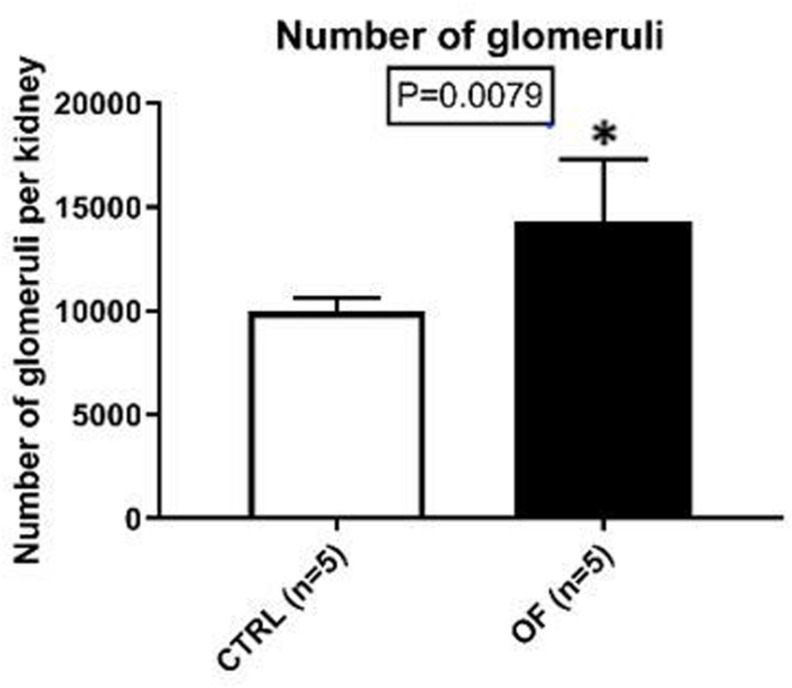
Mean glomerular count per kidney in control (CTRL) and overfed (OF) groups at adulthood. Results are expressed as means ± SD, *p < 0.05.

Glomerular number was assessed at adulthood. Glomerular count in kidneys of postnatally overfed animals was 44% higher than controls (*n* = 5 in each group, *p* = 0.0079) (Figure 3).

### Senescence Associated β-Galactosidase Activity

**FIGURE 4:**
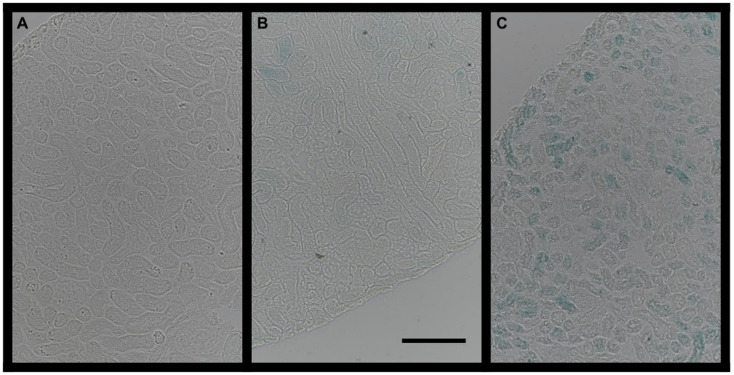
β-Galactosidase staining in three different index animals (**A**, negative control; **B**, CTRL group; **C**, OF group). The bar on picture B represents 50 μm.

β-Galactosidase staining, reflecting senescence associated β-Galactosidase activity, was more widely distributed and more intense in early overfed animals, as shown in Figure 4. These findings indicate a higher number and larger distribution of senescent cells at weaning in the kidneys of animals who underwent early postnatal overfeeding.

### Senescence Pathways Analysis

**FIGURE 5:**
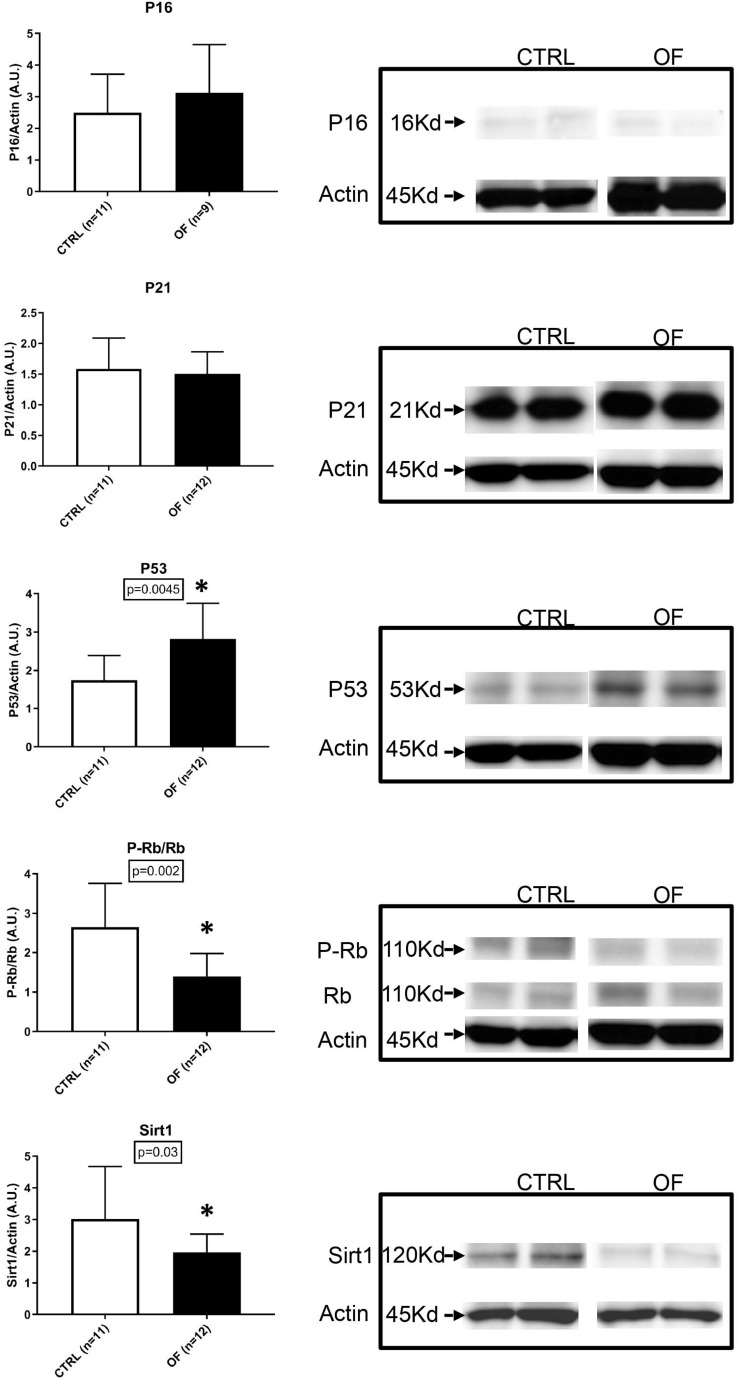
Protein expression of P-Rb, Rb, P53, P16, P21, and Sirt1 were analyzed in the kidney of control (CTRL) or overfed (OF) mice at weaning. P53 protein expression was significantly increased in OF group (*p* = 0.0045) while Sirt1 protein expression and P-Rb/Rb ratio were significantly decreased (*p* = 0.03 and 0.02, respectively) compared to the CTRL group. Results are expressed as mean ± SD, *p < 0.05.

**FIGURE 6:**
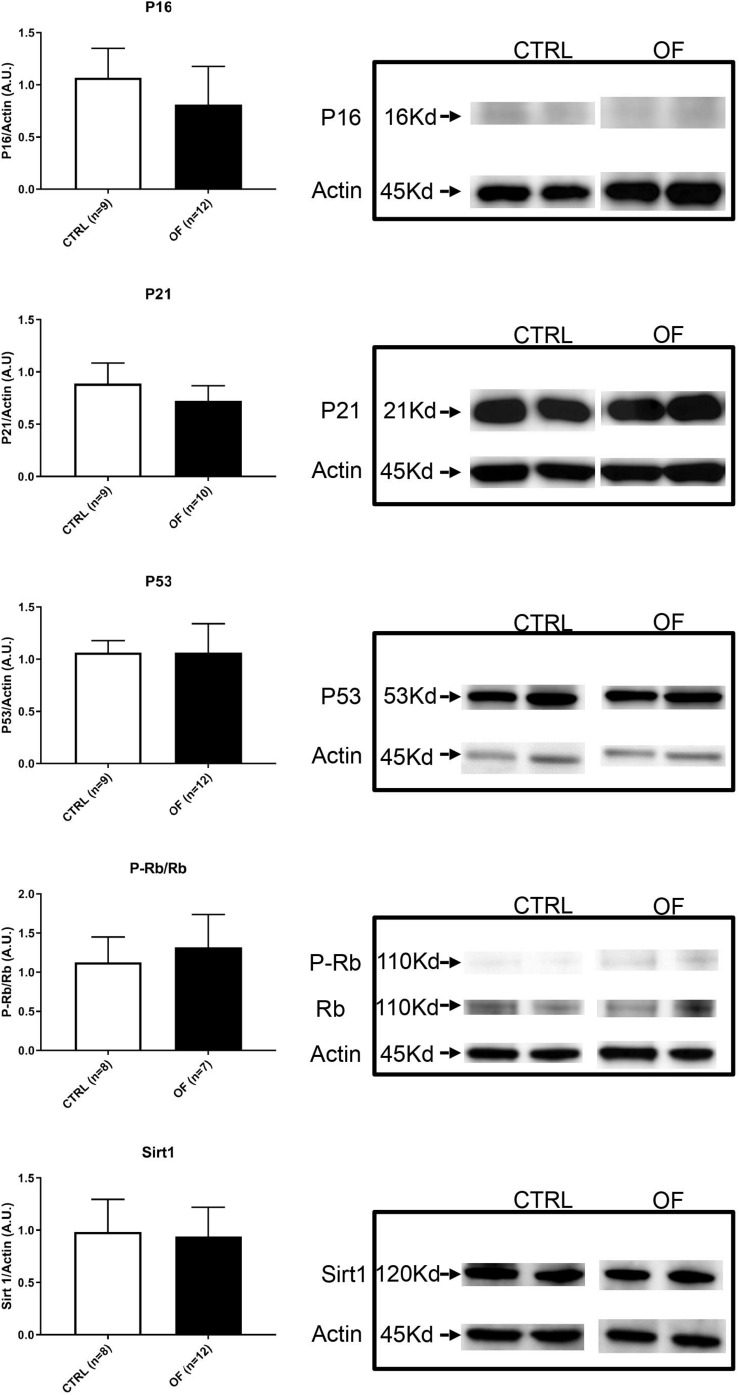
Protein expression of P-Rb, Rb, P53, P16, P21, and Sirt1 were analyzed in the kidney of control (CTRL) or overfed (OF) mice at adulthood. No significant change in protein expression in any of the analyzed markers was observed between CTRL and OF group. Results are expressed as mean ± SD, *p < 0.05.

To further investigate the underlying mechanisms involved in cell senescence after early postnatal overfeeding, we analyzed the protein expression of P-Rb, Rb, P53, P21, P16, and Sirt1. The higher number of senescent cells in the kidney after early postnatal overfeeding demonstrated by a higher β-Galactosidase staining, was confirmed by quantification of protein expression of factors involved in cellular senescence pathways at weaning (Figure 5). The final effector Rb was found to be significantly hypophosphorylated in the OF group compared to control group at weaning (*n* = 11 for CTRL, *n* = 12 for OF, *p* = 0.002), indicating that larger parts of the analyzed tissue have entered a state of senescence. Activation of senescence pathways was further confirmed by a significant increase in protein expression of P53 (*n* = 11 for CTRL, *n* = 12 for OF, *p* = 0.0045). Protein expression of P16 and P21 were not significantly different between groups (*n* = 11 for CTRL, *n* = 9 for OF, *p* = 0.46, *n* = 11 for CTRL, and *n* = 12 for OF, *p* = 0.83; respectively). Furthermore, the protein expression of longevity-promoting factor Sirtuin 1 (Sirt1) was found to be significantly decreased in the OF group (*n* = 11 for CTRL, *n* = 12 for OF, *p* = 0.03) compared to control group (Figure 5). No significant difference in expression of the analyzed factors was observed at adulthood (for P16: *n* = 9 for CTRL, *n* = 12 for OF, *p* = 0.11; for P21: *n* = 9 for CTRL, *n* = 10 for OF, *p* = 0.11; for P53: *n* = 9 for CTRL, *n* = 12 for OF, *p* = 0.35; for P-Rb/Rb: *n* = 8 for CTRL, *n* = 7 for OF, *p* = 0.61; and for Sirt1: *n* = 8 for CTRL, *n* = 12 for OF, *p* = 0.95) (Figure 6).

## Discussion

Increasing number of evidence suggests that the postnatal period is a critical window of sensitivity for long-term kidney health. Indeed, when applied during this period, a transient overnutrition leads to increased body weight, elevated BP, and increased glomerular number (Boubred et al., 2007, 2009; Yim et al., 2012, 2013, 2014). Consistent with those findings, animals exposed to postnatal overfeeding in our study exhibited increased body weight, elevated BP at adulthood, and increased glomerular number. Our results further shed new light on the role of senescence pathways in the kidney after transient early postnatal overfeeding.

We observed that early transient postnatal overfeeding in mice is associated with an early activation of senescence pathways in the kidney. This increase has been evidenced by the semi-quantitative assessment of tissular senescence by β-Galactosidase staining and confirmed by Western blotting. Indeed, the pathway’s end effector Rb is significantly hypophosphorylated at weaning in OF animals, thus indicating larger parts of the analyzed tissue have entered a state of senescence. We also found P53, an upstream regulator of Rb, to be significantly increased after early postnatal overfeeding.

We found that the longevity-promoting factor Sirt1 is significantly downregulated at weaning in the kidney of animals who underwent early postnatal overfeeding. This is consistent with the ongoing overfeeding at that time point, as Sirt1 expression is regulated by the intracellular energy balance (Oellerich and Potente, 2012). Furthermore, Sirt 1 interacts with P53, as these factors inhibit each other (Kong et al., 2015). Thus the decrease of Sirt1 levels at weaning is consistent with P53 upregulation. It is noteworthy that the protective effects that Sirt1 exerts on the kidney are numerous and interact with many cellular pathways (Kong et al., 2015). Sirt1 downregulation is likely to contribute to programming of renal disease after early transient postnatal overfeeding. In rodents, nephrogenesis begins in the middle gestation and continues for several days after birth. It is likely that overfeeding during the early postnatal key window is responsible for the increased nephron number. However, reduced glomerular volume in this context has been observed, which can explain the later development of glomerulosclerosis and renal failure. Our study focused on the molecular mechanisms underlying the previously described renal sequellae of postnatal overfeeding and demonstrate that senescence pathway are activated early in this model. Several studies in humans and animal models demonstrate the presence of senescent cells in multiple localizations of the kidney (cortical tubules, glomeruli, interstitium, and arteries) in the context of renal aging (Valentijn et al., 2018) and diseases, such as deoxycorticosterone acetate (DOCA)-salt-induced hypertension, streptozotocin-induced diabetic nephropathy, and cisplatin-induced nephrotoxicity (Westhoff et al., 2008; Kitada et al., 2014). In this study, we show that senescence pathways may also play a role in the programming of renal alterations after early transient postnatal overfeeding. Interestingly, Jennings et al. (1999) demonstrated that early postnatal growth impacts longevity in male rats and affects telomere shortening in the kidney, conditions known to be associated with senescence.

Importantly, these alterations in senescence pathways and Sirt1 expression in the context of transient postnatal overfeeding do not seem to be specific to the kidney. Our group previously showed similar alterations in the liver at adulthood in the same animal model (Yzydorczyk et al., 2017). However, none of the alterations in protein expression described at weaning in the kidney were observed at adulthood. The kidney is a non-regenerative organ, and kidney damage secondary to transient and early exposure to harmful factors may induce definitive organ damage. Even if the noxious stimulus is removed, the already caused renal harm cannot be corrected, as no additional glomeruli can be formed later in life, after weaning. This will affect renal health in the long term, although the noxious stimulus is not present anymore. In this model, overfeeding was induced during lactation by litter size reduction, with a significant impact on body weight, blood pressure and glomerular count later in life, and associated with early modifications in senescence pathways. In humans, breastmilk is beneficial compared to formula for infant nutrition, with a significantly decreased systolic and diastolic blood pressure, and a significantly lower BMI at adulthood (Fergusson et al., 2014). This study is a reminder that early postnatal overfeeding, including during lactation, still entails long-term risks.

This study presents several limitations. Evaluation of long-term renal consequences was limited to glomerular count and blood pressure measurement. Further studies should aim at studying in parallel the molecular changes in the senescence pathways and other parameters of late renal dysfunctions, such as renal function and glomerulosclerosis. In addition, to confirm the causality of senescence pathway activation and decrease of Sirt 1 expression on later renal dysfunction, further studies should investigate if Sirt 1 induction using resveratrol (Ligi et al., 2011; Vassallo et al., 2014) or blocking of selected factors involved in senescence pathways, such as P53, could reverse the activation of identified senescence pathways and prevent the development of renal dysfunction at adulthood. Another limitation of this study is that only male animals were analyzed. However, future studies exploring the response to early postnatal overfeeding should be performed in females as well.

## Conclusion

In conclusion, senescence pathways are upregulated in the mouse kidney after early postnatal overfeeding secondary to litter size reduction, and may contribute to programming of renal disease in adulthood. Given the growing interest in early prevention of renal disease (Luyckx et al., 2017; The Low Birth Weight and Nephron Number Working Group, 2017), it can be speculated that optimizing nutrition early in life may contribute to reducing the risk of CKD at adulthood.

## Data Availability Statement

Western blots generated for this study are included in the supplementary data can be found on https://doi.org/10.5281/zenodo.3740464. Other datasets generated for this study are available on request to the corresponding author.

## Ethics Statement

The animal study was reviewed and approved by the Comité d’Ethique de l’Expérimentation Animale, Université de Bourgogne, Dijon, France, protocol agreement number: 3710.

## Author Contributions

US and HC contributed to the conceptualization and project administration. US, CV, CY, BS, and HC contributed to the methodology. CJ, CY, BS, and KN contributed to the investigation. CJ contributed to the formal analysis and the original draft preparation. CJ, US, CV, CY, BS, KN, J-BA, MB, FC, and HC contributed to the review and editing. US, FC, and HC contributed to the supervision. US, CV, and HC contributed to the resources. CJ, US, CY, BS, J-BA, and HC contributed to the funding acquisition.

## Conflict of Interest

The authors declare that the research was conducted in the absence of any commercial or financial relationships that could be construed as a potential conflict of interest.
